# Pathogenesis of Uveitic Glaucoma

**DOI:** 10.5005/jp-journals-10028-1257

**Published:** 2018

**Authors:** Dimitrios Kalogeropoulos, Velota CT Sung

**Affiliations:** 1,2 Birmingham and Midland Eye Centre, Sandwell and West Birmingham Hospital, NHS Trust, UK

**Keywords:** Glaucoma, Intraocular pressure, Ocular hypertension/hypotony, Ocular inflammation, Uveitis, Uveitic glaucoma

## Abstract

Uveitic glaucoma consists one of the most serious complications of intraocular inflammation and, despite its rarity, is considered as one of the leading causes of preventable loss of vision worldwide. It has been associated with a wide spectrum of inflammatory diseases, but its pathogenesis is still not fully comprehended. It appears that the type of inflammation, the steroid-response and the anatomical alterations of the anterior chamber play a pivotal role. To our knowledge, the mechanisms may be both acute and chronic. The main targets of the treatment are to control the inflammation and reduce the intraocular pressure (IOP). The management of glaucoma associated with uveitis remains an extremely challenging condition for ophthalmologists. The successful treatment of uveitic glaucoma is inextricably correlated with prompt and immediate therapeutic decisions. Very often a solid collaboration between clinicians from different specialties may be required for treating the underlying disease.

Further understanding of its pathogenesis can indicate therapeutic targets and may lead to the development of new and more efficient therapeutic approaches. New glaucoma surgical modalities may ameliorate the prognosis after surgical intervention, but this calls for further evaluation. This study aims to highlight the complexity of uveitic glaucoma analyzing the main pathogenetic mechanisms and the correlations with the inflammatory response.

**How to cite this article:**

Kalogeropoulos D, Sung VCT. Pathogenesis of Uveitic Glaucoma. J Curr Glaucoma Pract 2018;12(3):125-138.

## INTRODUCTION

It has been estimated that uveitis affects more than 2 million people worldwide and approximately 10% of them will suffer from irreversible visual loss due to the complications of the disease that include damage to the retina and glaucoma.^1,2^ Uveitic patients have a high risk of developing glaucoma, which is attributed not only to the uveitis itself but also to the corticosteroids used for the treatment of uveitis treatment. Uveitic glaucoma is defined as a raised IOP in a patient with uveitis who is diagnosed with optic nerve damage causing typical progressive visual field loss.^2,3^ Interestingly, raised IOP in uveitis can be recorded in patients with either an open or a closed angle. However, the pathophysological mechanisms of uveitic glaucoma are likely to differ among the wide spectrum of types and causes of uveitis. For instance, acute uveitis (e.g., herpetic uveitis, toxoplasmosis, and Posner-Schlossman syndrome) is characterized by an acute elevation of IOP, whereas other types of uveitis with a chronic course (e.g., HLA-B27 related anterior uveitis) can present with a gradual increase in IOP over time. However, some may present with high IOP that gradually decreases (hypotony) over time (e.g., in Fuchs’ heterochromic cyclitis and Juvenile idiopathic arthritis).^4^ The pathogenesis of uveitic glaucoma is characterized by several complex biochemical and cellular processes in the aqueous together with macroscopic morphological alterations in the anterior chamber.^5^ It is hard to dispute that the management and the long-term control of uveitic glaucoma consist of challenging clinical entitles that calls for anti-inflammatory and IOP-lowering medications and more often than not, surgical interventions.

## PATHOGENETIC MECHANISMS OF UVEITIC GLAUCOMA

The mechanisms by which ocular inflammation causes an increase of the IOP are not integrally comprehended, and many of the relevant pathways and features are still covered with a veil of mystery.

In contrast with primary glaucoma, where pressure-independent mechanisms may be related, uveitic glaucoma is usually correlated with increased IOP, although the elevation of IOP may happen intermittently. These patients are usually younger with acute elevation of the IOP and fluctuating responses to the antiglaucomatous drugs.^6^ However, a drop of the IOP may occur during an attack of acute uveitis because of a reduction in the aqueous secretion, secondary to ciliary body inflammation, and an increase in uveoscleral outflow.^6^ The eyes of uveitic patients are defined by complex interactions between the angles (open or closed), trabecular outflow, fluctuations in the aqueous production and the response to steroids. Regarding the elevation of IOP in these individuals, it is mostly attributed to the increased outflow resistance, which distorts the equilibrium between aqueous production and outflow.^3^ As for the eyes with closed angle uveitic glaucoma, three mechanisms have been described (1) formation of posterior synechiae between the iris and lens causing seclusio pupillae, (2) formation of peripheral anterior synechiae between the iris and the cornea and, (3) forward rotation of the ciliary body.^3^

The wide spectrum of variations in the underlying trabecular function in different individuals also adds to the perplexity of the uveitis. It is expected that the trabecular meshwork function may be affected while aging and therefore older patients are more susceptible to intraocular inflammation in comparison with younger individuals.^7,8^ Interestingly, older age consists of a risk factor for increased IOP in patients with uveitis. Probably, the accumulation of pathological alterations secondary to the chronic inflammatory activity may also be relevant to the IOP rise in older uveitic patients.^9^ Interestingly, the management of uveitic glaucoma in our patients appears to be more difficult and challenging in the younger age group. Whereas, younger individuals may have a stronger optic nerve that can withstand high pressure for a more extended period of time, it appears that older patients develop severe optic nerve damage even during shorter intervals of raised IOP and consequently more visual disabilities.

## FACTORS AFFECTING INTRAOCULAR PRESSURE CHANGES IN UVEITIS

Ocular hypertension (OHT) and hypotony in uveitic eyes can potentially lead to blindness. It has been reported that 10 to 46% of uveitic patients develop increased IOP, but secondary glaucoma occurs usually in patients with chronic uveitis and those with Posner-Schlossman syndrome, Fuch's heterochromic iridocyclitis or in those with HSV/VZV-related uveitis.^10,11^ Increased eye pressure in patients with uveitis has been associated with poor visual outcomes.^12^

Hypotony is less common in uveitic eyes; it has been recorded in up to 10% of patients and is most prevalent in children, especially in those with juvenile idiopathic uveitis.^13^ Furthermore, in patients with uveitic glaucoma, there is also a higher propensity for postoperative hypotony due to the impairment of the ciliary body caused by the chronic and relapsing nature of the intraocular inflammatory activity. Similarly, with OHT, hypotony in the context of uveitis is associated with poor visual outcomes in both adults and children.^13^

A relevant study by Aman et al. assessed OHT and hypotony as determinates of outcomes in uveitis in a retrospective, observational study of 442 uveitic patients. They found that OHT was related to the anterior uveitis, increasing age, and poor presenting visual acuity. Hypotony was more common in anterior uveitis than in non-anterior uveitis. Moreover, fluctuations in IOP appear to be an important parameter that leads to visual impairment in these patients.^14^

### Factors Reducing Intraocular Pressure

Specific types of uveitis, such as those related to HLA-B27 might present with acute hypotony. Interestingly, there are many factors that can lead to decreased IOP in uveitis. Out of them, two of the most important are the increased uveoscleral outflow and the reduced production of aqueous humor. Several studies in animals,^15–17^ and humans^18–21^ have concluded that the production of aqueous humor declines while the uveoscleral outflow raises, resulting in hypotony. Other studies have explored the chronic effects of ocular inflammation, which may be correlated with the pathogenesis of uveitis in JIA and Fuch's heterochromic cyclitis. There is evidence that vascular permeability may be elevated over the course of time after an episode of acute inflammation,^22^ leading to increased aqueous production.^3^

More information about the uveoscleral outflow derives from investigations on prostaglandins. It has been shown, that prostaglandins reduce the IOP by increasing the uveoscleral outflow.^19^ Hence the prostaglandin analogs are the most frequently used eye drops in the treatment of glaucoma.^23^ Older studies have highlighted that the ocular levels of prostaglandins increase in uveitis explaining probably why there is also an increase in the uveoscleral outflow. More specifically, Eakins et al. detected elevated levels of prostaglandins in the eyes of uveitis patients that were receiving no treatment, in comparison with the excessively low levels in those treated with steroids, and none in non-uveitic patients and patients with cataract.^24^ The decrease of prostaglandin levels and the following decrease in uveoscleral outflow could potentially reflect a pathogenetic mechanism by which steroids cause glaucoma in steroid responders.^4^

Interestingly, it appears that diclofenac, which is a non-steroidal anti-inflammatory drug, reduces the flare in the anterior chamber after laser trabeculoplasty, through the inhibition of prostaglandin synthesis.^25^ It has been reported that apart from the uveoscleral outflow, prostaglandins can also increase the aqueous production and trabecular outflow.^24,26^

In acute uveitis, there is an increase in the uveoscleral outflow and probably the permeability of the blood-aqueous barrier. If aqueous production declines and uveoscleral outflow increases in uveitic patients, then to develop elevated IOP, either trabecular outflow must drop or there must be different pathogenetic mechanisms for chronic intraocular inflammatory processes.^4^ In addition, it has not been defined yet whether these alterations take place in types of uveitis with increased IOP.

### Factors Increasing Intraocular Pressure

The levels of aqueous proteins have been found to be elevated in uveitic patients, indicating that they may be associated with the trabecular outflow. Previous studies have investigated the role of aqueous proteins during acute intraocular inflammation.^7,27–31^ However, due to the fact that these studies have explored acute uveitis, it was not feasible to define the long-term effect of elevated aqueous proteins in uveitic eyes. It is a well-established knowledge that the protein concentration in the anterior chamber is increased in acute uveitis, causing a drop of trabecular outflow. This could probably happen more extensively in individuals with clinical entities that present with the acute rise of IOP.^4^ Though it must be underlined that in Posner-Schlossman syndrome, which presents with acutely raised IOP, the levels activity in the anterior chamber remain normally low, implying that other mechanisms (e.g., trabeculitis) contribute in the increased IOP.

The increased number of trabecular precipitates that include proteins, inflammatory cells, and debris in the anterior chamber of uveitic eyes may decrease trabecular outflow by clogging of the trabecular meshwork. Evidence that inflammatory cells lead to clogging of the trabecular meshwork derive from studies that recorded acute rises in IOP after Nd: YAG laser capsulotomy.^25^ Moreover, there is evidence that the acute inflammatory processes that follow Nd: YAG laser can also lead to chronic OHT, underlining that the clogging of trabecular meshwork that occurs during an episode of intraocular inflammation may have long-term effects on trabecular outflow and subsequently on IOP.^32^ Gonioscopy of the trabecular meshwork can reveal its obstruction by inflammatory precipitates in several pathological entities, such as in Grant's syndrome,^33^ pseudoexfoliations,^34^ and pigmentary glaucoma.^35^

The inflammation of the trabecular meshwork, which is known as trabeculitis, may also cause a rise in IOP. Herpetic uveitis consists one of the most characteristic examples. Two older studies by Hogan et al. and Townsend et al. have described the histological alterations caused in enucleated human eyes^36^ and rabbits^37^ with herpetic inflammation, respectively. Both studies highlighted that trabeculitis could play a critical role in the elevation of IOP in uveitis, especially when caused by HSV. However, it is yet to be defined whether trabeculitis interferes in types of uveitic glaucoma with a more chronic course, such as those related to Fuch's heterochromic cyclitis or juvenile idiopathic arthritis (JIA).

Interestingly, T-cells consist the largest cell population in the aqueous, vitreous, retina and the uveal tract of uveitic patients. More specifically, Th-1 cells might have a substantial contribution in the pathophysiology of uveitis, but their role in uveitic glaucoma remains uncertain.^38^ A study by Murray et al. investigated the aqueous humor obtained from patients with and without uveitis during a cataract extraction surgery. Individuals with uveitis demonstrated an inflammatory response mediated by T-cells. There was a prevailing expression of IL-2 and IFN-γ, which are Th-1 related cytokines and their levels were significantly lower in non-uveitic individuals.^39^ The dominant role of T-cells in uveitis has been also reported by other studies.^40,41^ Subsequently, T-cells are likely to consist the main immune cells in uveitis. However, there is no evidence at the moment to associate the presence of T-cells and the related cytokines to the risk of developing uveitic glaucoma. Moreover, the cytokine response may vary among different types of uveitis (i.e., infectious and non-infectious) and whether each type will present with acutely increased IOP (mainly in infectious uveitis) or hypotony.

Nonetheless, the levels of proinflammatory cytokines have been associated with the activity of inflammatory activity in various types of uveitis.^42–46^ Ohira et al. analyzed the effects of factors on the levels of aqueous humor proinflammatory cytokines and growth factors in uveitic eyes. According to the results mean interleukin (IL)-6, IL-8, monocyte chemotactic protein (MCP)-1, tumor necrosis factor (TNF)-α and VEGF were found to be higher in cases with uveitic glaucoma than those in cataract (non-glaucomatous) cases.^47^ Additionally, IL-6, MCP-1, and VEGF were all higher in uveitic glaucoma than in patients with POAG. The uveitic cases with a history of phacoemulsification indicated higher levels of IL-6, IL-8, MCP-1 and PDGF-AB/BB in comparison with the phakic eyes. Finally, the presence of cells in the anterior chamber was associated with higher levels of TNF-α, IL-8 and PDGF-AB/BB. As for PDGF-AB/BB level, it was found to be higher in infectious rather than in non-infectious uveitis.^47^

Specific enzymes, such as angiotensin-converting enzyme (ACE) and Rho-kinase may interfere in the pathogenesis of uveitic glaucoma. Typically, serum and aqueous ACE levels are increased in uveitis related to sarcoidosis.^48^ This feature, as well as the potential role of ACE-inhibitors, has been confirmed by several experimental studies.^49–51^ Rho-kinase regulates the formation of actin stress fibers, focal adhesion, and cellular contraction. A study by Rao et al. used Western blot analysis to find out that that there are increased levels of Rho-kinase in cells from human trabecular meshwork and Schlemm's canal.^52^

Similarly, another study reported that individuals with Behcet's disease present higher rates of Rho-kinase gene polymorphisms. Moreover, these patients are expected to have higher levels of Rho-kinase expression mRNA in their blood in comparison with healthy individuals.^53^ However, it must be cited that the role of the enzymes mentioned above has not been clarified completely yet.

Free radicals, which are produced by macrophages and neutrophils, have also been implicated in the pathogenesis of intraocular inflammation. In particular, they are known to cause peroxidation of lipid cell membranes and subsequently damage the retinal tissue.^54^ Despite the fact that increased concentrations of free radicals have been detected in the aqueous humor of glaucomatic patients, it is not certain of whether they contribute to uveitic glaucoma or not. Probably, they could play some role in the development of uveitic glaucoma by causing cumulative damages to the trabecular meshwork.^55,56^

Finally, an increase in the IOP related to the corticosteroids that are used for the control of inflammatory reactions in uveitis has been recorded in 18–36% of patients; these individuals are described as steroid responders.^57,58^ Clinically, a response to steroids is expected to develop within 2 to 6 weeks after starting therapy, but can potentially happen at any time. Apart from that, it is not always easy to distinguish when this occurs as a result of the steroids or the underlying inflammation. It appears that only 5% of the normal population demonstrates responsiveness to corticosteroids. However, the steroid response rates increase substantially due to the impairment of the conventional outflow.^59^ Especially, children are very susceptible to an increase of IOP related to steroids.^60, 61^ Shrestha et al. monitored and studied 116 consecutive new uveitic patients, recording the IOP at presentation, at 1 week, 3 and 6 weeks. They recorded that 20% of these eyes developed ocular hypertension, which was at a percentage of 64.5% was attributed to corticosteroids (37.03% of the oral group, 14.28% of the posterior sub-tenon group and 8.57% of topical group). The same study indicated that timely medical treatment might avert the necessity of early surgical intervention for the control of eye pressure.^62^ A relevant study by Salam et al. also showed that a substantial proportion (61.1%) of the IOP rise in uveitic patients was steroid induced. Most probably, this is a result of trabecular meshwork obstruction due to an increase in degradation products of extracellular matrix and edema of trabecular meshwork cells,^63,64^

Interestingly, as a response to decreased pressure gonocytes, which consist of fibro-elastic cells in the anterior chamber of the normal human eye secrete polymerized mucopolysaccharides. This results in swelling of the cells of the trabecular meshwork and a subsequent decrease in the trabecular outflow. To curtail these effects and reduce IOP hyaluronidase breaks down the polymerized mucopolysaccharides. However, the release of hyaluronidase may be restricted due to the use of steroids, causing inhibition of mucopolysaccharides depolymerization.^65^ Some studies have investigated the genetic background of steroid responders, showing that a gene that may play some role is responsible for a protein named Myocilin, which is produced by the cells of the human trabecular meshwork.^66^ Despite the fact that there are not any known Myocilin mutations associated with steroid responders, it appears that the cells of human trabeculum increase the production of Myocilin in response to the administration of dexamethasone.^67^ Consequently, although genes might contribute in defining steroid responders more studies are required to confirm this hypothesis.

## UVEITIC GLAUCOMA IN ASSOCIATION WITH OCULAR INFECTIONS

In the vast majority of cases, viral anterior uveitis presents with elevated eye pressure, mild anterior chamber reaction, and iris atrophy. The development of molecular diagnostic techniques (e.g., Polymerase Chain Reaction–PCR), has contributed in the diagnostic accuracy in such cases that were previously defined as “hypertensive uveitis”.^68^ Characteristically, active HSV, VZV, and CMV replication has been demonstrated during ocular inflammation.^69^ It has been estimated that the prevalence of increased IOP in individuals with herpetic anterior uveitis varies from 50 to 90%,^68^ whereas glaucoma in eyes with viral uveitis is approximately 13%.^70^ Interestingly, it has been reported that the mean IOP is very often higher in uveitis attributed to VZV and CMV compared to that related to HSV.^69^ Ebola virus has also been detected in the aqueous humor of a patient who was free of viremia for a period of 9 weeks. Despite that, he developed unilateral uveitis with increased IOP during his recovery. It has been suggested that active viral replication may have a direct cytopathic effect, causing intraocular inflammation.^71^ The eye consists of an anatomical site defined by the so-called “immune privilege”, implying that viruses can survive, replicate and “elude” from the systemic immune response.

There is recent evidence suggesting that there might be a correlation between CMV infection and clinical entities, such as Fuchs’ heterochromic cyclitis and Posner–Schlossman syndrome.^72,73^

## MAJOR INFLAMMATORY CLINICAL ENTITIES OF THE EYES AND THEIR ASSOCIATION WITH SECONDARY GLAUCOMA

Twenty percent of all patients with chronic uveitis will develop glaucoma. As it has been already mentioned, the clinical course and manifestation of uveitic glaucoma vary depending on the etiologic factor.^74^ The cause of uveitis differs among different ethnic groups and may also vary even among regions of the same country.^75^ The higher rates are recorded in patients with HSV keratouveitis (54%), VZV uveitis (38%), sarcoidosis (34%), rheumatoid arthritis-related iridocyclitis, Fuchs heterochromic iridocyclitis (27%), Posner-Schlossman syndrome,^74^ juvenile-idiopathic arthritis (JIA), Lyme-associated uveitis, cancer-associated uveitis,^76^ syphilis, Behcet's disease, sympathetic ophthalmia and pars planitis.^74^ In many of these diseases, some specific clinical features can be very helpful in the establishment of a precise diagnosis. Some of the most characteristic signs are described below.

### Fuchs’ Heterochromic Iridocyclitis

Fuchs’ heterochromic iridocyclitis (FHIC) consists an idiopathic, chronic, low-grade iridocyclitis with heterochromia (which is attributed to the iris stromal atrophy). It is typically unilateral and affects the hypochromic eye, but it has been reported that in 13% of cases there is a bilateral involvement.^77^ Unfortunately, it remains undiagnosed in many cases, because heterochromia may be subtle and not be easily detected. The age of onset is between 20 to 40 years and both genders appear to be equally affected.^78^ This rare type of uveitis presents with diffuse, fine and scattered keratic precipitates (KP). KPs are stellate-shaped with fine filaments between them. Clinical findings also include iris atrophy and patchy transillumination due to the loss of pigment epithelium.^79^ Interestingly, an association with rubella virus has been reported.^80,81^ A gonioscopy is expected to show an open angle and features of fine neovascularization, which may cause bleeding.^82^ The histological findings include hyalinization of blood vessels, anterior stromal depigmentation, cellular infiltrations, and Russell bodies. The trabecular meshwork is infiltrated by mononuclear inflammatory cells (i.e., lymphocytes and plasma cells), causing trabeculitis, rubeosis and collapse of the Schlemm's canal with subsequent IOP increase.^79^ Interestingly, iris angiography can detect leakage of the iris vessels and ischemic alterations of the iris. The most common complications are secondary glaucoma and posterior subcapsular cataract, but synechiae are not a typical complication in FHIC. The incidence of glaucoma may vary from 13–59% and usually develops with time. It consists the major cause of vision loss in patients with FHIC. Although it is generally intermittent and initially well controlled, it can become complicated and chronic.^1^ Glaucoma is more often in Africans and patients with bilateral involvement.^77^ In most of the cases, the glaucoma is persistent, even after the uveitis has subsided, and does not respond to steroids. FHIC is considered to be a benign ocular disorder and does not call for therapy. Treatment with steroids may only accelerate the development of cataract and IOP elevation. In terms of medication, aqueous suppressants can be effective in the initial glaucoma control and in long term basis surgical treatment may be necessary.^1^

### Posner-Schlossman Syndrome (Glaucomatocyclitic Crisis)

Posner-Schlossman syndrome typically manifests with recurrent episodes of unilateral mild cyclitis and heterochromia, affecting individuals aged between 20 to 60 years old. Although the inflammatory activity may be mild, IOP may elevate up to 40 to 70 mm Hg during an acute episode. This rise in the eye pressure has been correlated with the aqueous levels of prostaglandins and usually resolves spontaneously.^83^ The pathogenetic mechanisms have not been clarified yet. Some of the possible etiologic factors include HSV and CMV infections,^84,85^ an immunogenetic factor that involves HLA-Bw^54^^,86^ gastrointestinal disease and several allergic factors (i.e., eczema, urticaria, asthma, rhinitis, contact dermatitis, angioneurotic edema, intolerance to aspirin and food allergies).^87^ The prognosis is benign, with only exception the patients that develop glaucomatous alterations, which is recorded in about 25% of cases. Clinical findings include small, flat, fine, non-pigmented KP detected in the inferior corneal endothelium. In gonioscopy, the angle appears to be open with some occasional trabecular precipitates.^88^ The therapeutic approach includes steroids and antiglaucoma medication (beta-blockers and carbonic anhydrase inhibitors).^82^ It has been shown that oral indomethacin, prostaglandin inhibitors and subconjunctival polyphloretin (prostaglandin antagonist) are effective in lowering the IOP during acute attacks.^83,89^ Surgical treatment is indicated in patients that glaucoma persists after maximal medical treatment.

### Herpetic Uveitis

The development of secondary glaucoma consists the most common complication of herpetic uveitis.^90^ It has been reported that 28–45% of patients with HSV keratouveitis demonstrate transient increased IOP and 10–54% may present with uveitic glaucoma.^90,91^ Active iridocyclitis accompanied by an acute increase in eye pressure are the main features of herpetic infection and in most cases, HSVs or VZVs are the etiologic factors. These spikes in the IOP occur as a result of the trabecular meshwork inflammation, similarly to hypertensive episodes of Posner-Schlossman syndrome that has been described above.^92^ This explains why the IOP returns to normal levels while responding to topical corticosteroids. It must be underlined though, that the elevated IOP can occur secondarily to the obstruction and swelling of the trabecular meshwork. Episodes of herpetic uveitis are typically unilateral, acute and in severe cases may present with hyphema, hypopyon, fibrin deposition and formation of anterior synechiae.^93^ The management of glaucoma in these patients might be complicated due to the explosive nature of the disease and the potential recurrences. In many cases, long-term antiviral prophylactic treatment is required to avert recurrences.^94^

### Juvenile Idiopathic Arthritis

Juvenile idiopathic arthritis (JIA) is an autoimmune disorder that affects children younger than 16 years old and typically lasts for more than 6 months.^74^ The JIA-related uveitis is usually a non-granulomatous, bilateral, asymptomatic anterior uveitis, which is in most cases preceded by arthritis.^95^ The prevalence of secondary glaucoma in these patients has been reported to vary from 14–27%.^95–9^^7^ However, a relevant study indicated a higher prevalence of 42% of glaucoma or OHT related to JIA uveitis.^1^ It has been shown that individuals with persistent low-grade uveitis are at a higher risk of developing glaucoma. JIA-related glaucoma often occurs with open angles, but secondary angle-closure caused by pupillary block as a result of the formation of posterior synechiae is relatively common.^98^ Apart from glaucoma, the main complications that can lead to loss of vision are a cataract, band keratopathy, and cystoid macular edema.^99^ Regarding medication, the therapeutic scheme includes a topical steroid, cycloplegics that may be followed by systemic steroid therapy and possibly regional injection of steroids. In persistent and more severe cases immunomodulation (e.g., methotrexate) can be incorporated in the management of the disease, resulting in low toxicity and high efficacy. According to recent studies adalimumab, which is an anti-TNF-α agent, has shown efficacy in treating refractory uveitis in multiple settings, including juvenile idiopathic arthritis.^100–103^ With regard to the treatment of glaucoma patients are initiated on antiglaucoma medications, but in complicated and severe cases surgical treatment (e.g., trabeculectomy or tube shunt surgeries) may be unavoidable. Unfortunately, many of them might require medication even after surgery.^1,98^

### HLA-B27-related Anterior Uveitis

Ankylosing spondylitis usually occurs around the 2nd to 3rd decade of life and mostly in HLA-B27-positive Caucasian males. The pragmatic etiology and the pathogenetic mechanisms are not fully understood, and its diagnosis may be difficult and complicated. Acute anterior uveitis (AAU) is the most universal extra-articular manifestation and occurs in approximately 20–30% of individuals with ankylosing spondylitis.^104^ Almost half of the uveitis cases are related to HLA-B27 antigen. Uveitis can be the very first manifestation an undiagnosed rheumatic disease. Most of the times it has a good prognosis and responds to treatment.^104^ In these patients, AAU is defined as recurrent non-granulomatous iritis or iridocyclitis of acute onset, the duration of which is shorter than 3 months but can become chronic with multiple sequelae^105,106^ symptoms and signs include pain, photophobia, hyperemia, lacrimation, and blurred vision. A large number of cells and flare, keratic precipitates (never of the mutton-fat type) and edema of the iris with posterior synechiae can be observed.^106^ Additionally, a typical feature is that there is no simultaneous AAU of either eye, which can be alternating.^107^ In the severe case, even if the inflammatory process has already been controlled, a chronic and irreversible breach in the blood-aqueous barrier can happen, accompanied by an elevation in protein levels. However, this is not regarded as a parameter of therapeutic failure or relapse.^108^ As expected, the chronic course of the disease may cause various complications, mainly in HLA-B27-positive patients. These include low visual acuity, cataract, seclusion, and pupillary occlusion, secondary glaucoma, cystoid macular edema (CME), folds of the internal limiting membrane of the retina and macular hole.^106^ Interestingly, AAU of prolonged and uncontrollable course is considered as a risk factor for the progression of uveitis to the posterior pole causing further ocular manifestations.^109^

### Sarcoidosis

Sarcoidosis is a multisystem disease that affects young people aged between 20 to 40 years old and has a higher prevalence in females.^110^ The main ocular pathological findings comprise panuveitis, snowballs, phlebitis, candle wax dripping, mutton fat KP, Bussaca and Koeppe's nodules.^111^ As it has already been mentioned above the underlying mechanism of the IOP elevation in uveitis comprises disruptions in the ciliary process and blood-aqueous barrier, allowing inflammatory-mediated cells (e.g., neutrophils and T-cells) and plasma protein to enter the anterior chamber and subsequently causing an increase in outflow resistance. Additionally, mechanical flow disorders, such as peripheral anterior synechiae (PAS), which are related to the formation of granulomas, lead to structural alterations in the pathway of aqueous outflow. Moreover, inflammatory debris and nodules, detected in gonioscopy, can lead to obstruction of the trabecular meshwork and subsequent rise of the IOP.^111,112^ These pathological changes can lead to secondary angle closure glaucoma requiring surgical treatment, which can be extremely challenging in these patients due to the high risk of postoperative inflammation and subsequent surgical failure.^112^ Treatment with a systemic and topical corticosteroid can rapidly settle the pan-uveitis quickly but lengthy course of steroid treatments can lead to steroid-induced glaucoma and cataract formation.^57,58,63^ Cystoid macular edema is also a common complication of this condition that can lead to severe visual loss.^113^

### Syphilis

It has been estimated that 15% of syphilitic patients will develop interstitial keratitis, out of which 96% of cases are bilateral. It must be cited that 20% of the individuals with interstitial keratitis are expected to develop secondary glaucoma (either open or closed type).^114^ The open angle type of syphilis-related secondary glaucoma is characterized by a deep anterior chamber and no obvious signs of inflammatory activity. Glaucoma manifests at a later stage with elevated pressure. Irregular pigmentation may be found in the anterior chamber, accompanied by several degrees of synechiae formation. Older peripheral anterior synechiae that do not correlate with the levels of outflow obstruction can also be observed.^114^ The main mechanism is the endothelialization and hyaline membrane formation of the angles. This type of glaucoma is usually poorly responding to antiglaucoma medications and calls for surgical management.

With regard to the closed angle type, the anterior chamber is narrow and defined by a small anterior segment that could be indicative of interstitial keratitis in early infancy.^115^ In these cases, peripheral iridectomy can be of benefit in eyes without extensive peripheral synechiae.^114^ In eyes with permanent anterior synechiae surgical goniosynechialysis and filtering surgery seem to be necessary in the majority of cases.

### Vogt-Koyanagi Harada Syndrome (VKHS)

The VKHS usually manifests as granulomatous bilateral panuveitis, accompanied by central nervous and dermatologic pathological findings. Various human leukocyte antigen (HLA) correlations have been detected in patients with VKH disease, including HLA-DR4, HLA-DR53, and HLA-DQ4.^116–120^ Four distinct clinical stages have been described in VKH disease; these are the prodromal stage, uveitic stage, chronic stage, and recurrent stage. The anterior segment manifestations include granulomatous or non-granulomatous anterior uveitis. Additionally, mutton-fat keratic precipitates, Busacca nodules, and Koeppe nodules are typical features of granulomatous anterior uveitis Posterior synechiae may also be observed, especially in chronic cases, and pupillary membrane formation is quite common. Glaucoma can be present in 18 to 38% of cases and is mainly attributed to closed-angle mechanism secondary to extensive peripheral anterior synechiae formation. It consists of an extremely challenging condition because edema and anterior rotation of the ciliary body may occur and these cases are unlikely to respond to iridotomy.^6^

### Grant's Syndrome

This clinical entity is defined by an acute bilateral onset in patients aged above 50 years of life, which are asymptomatic in the vast majority of cases.^121,122^ One of the main features of Grant's syndrome is the gray-yellowish inflammatory precipitates of the trabecular meshwork. Interestingly, there is an excellent response to topical steroids, but antiglaucoma medication appears to be ineffective. Most commonly, the eye pressure gets back to a normal level within a couple of weeks after the initiation of topical steroid treatment that diminishes the inflammatory precipitates. Peripheral anterior synechiae may lead to severe angle closure, and a filtration surgery may be required.

### Scleritis and Episcleritis

Scleritis is a type of ocular inflammation that typically presents with pain and may involve the posterior or the anterior segment of the eye.^123^ Typically, it affects patients aged between 40 to 60 years old. As a matter of fact 12 to 46% of the individuals with posterior scleritis present with acute elevated pressure.^124^ Interestingly, acute angle closure glaucoma can be the main feature of scleritis, because of choroidal effusion and subsequent detachment. Uveitis, trabeculitis, pupillary block glaucoma, primary open angle, and neovascular glaucoma consist of some of the main pathogenetic mechanisms for the IOP rise.^125^ The therapeutic modalities include antiglaucoma medications, systemic anti-inflammatory treatment, and laser iridotomy when there is a pupillary block.

Similarly, it has been estimated that in episcleritis 4% of the patients will be diagnosed with secondary open-angle glaucoma either due to the inflammatory processes or due to the use of steroids.^126^

### Postoperative Uveitic Glaucoma

Uveitic glaucoma can occur after a complicated cataract extraction surgery. In this case, secondary glaucoma happens as a result of retained nuclear or cortical lens fragments. The pigment dispersion and the increase of the IOP are determined by malposition or subluxation of an intraocular lens. The so-called uveitis-glaucoma-hyphema syndrome is the most well-described clinical manifestation in patients with an AC-IOL or sulcus fixated IOL, and the explanation of the IOL may be necessary for some patients.^127^

## DIAGNOSTIC APPROACH

The substantial improvements and development of more sophisticated techniques in a clinical assessment of the optic nerve and retinal nerve fiber layer (RNFL), such as the optical coherence tomography (OCT), and of the angle, such as anterior segment OCT and ultrasound biomicroscopy (UBM) have been extremely important in the uveitic glaucoma in a similar way as to other types of glaucoma.

It appears that higher pressure levels related with uveitic glaucoma may lead to optic nerve structural damage that disappears when the pressure returns to normal levels.^128^ On the contrary, in uveitic eyes, the IOP is often excessively elevated despite the existence of only mild pre-perimetric optic disc changes.^74^ Therefore, apart from the clinical examination and the evaluation of the optic disc, it is critical to perform regular visual field tests to monitor closely any possible changes in the peripheral vision that could be indicative of glaucomatous progression. Imaging of the optic disc is a very useful modality for monitoring the glaucoma status, but it must be cited that uveitis consists of a significant confounding factor in the evaluation of RNFL thickness. Furthermore, it must be ensured that media opacification does not impede image acquisition. A study by Moore et al. showed noticeable thickening of RNFL in individuals with active uveitis and a thicker (than expected) RNFL in eyes with uveitic glaucoma,^129^ which could be related to the increased production of prostaglandin analogs and the breakdown of the blood-retinal barrier. Interestingly, after the curtailment of inflammatory activity, there is a decrease in retinal thickness, increased cupping and thinning of the RNFL.^130^ Therefore, “normal” measurements of the RNFL thickness in uveitic patients must be interpreted with a degree of skepticism, especially in those with increased IOP. The gradual resolution of edema of the RNFL may cause increased cupping and continuous thinning of the RNFL. It is suggested that screening for RNFL alteration related to glaucoma in uveitic patients must be carried out only during quiescent periods. A glaucomatous damage measurement can be considered as reliable when there is a thinning of the inferior quadrant. Early signs of damage that precede the disc or visual field alterations are an indication for more aggressive treatment.^131^ The OCT is also considered as a gold standard for setting a diagnosis of macular edema.^132^

UBM and anterior segment OCT are very useful in the assessment of the angle when the cornea is not sufficiently clear.^1^ Moreover, UBM contributes to the evaluation of various mechanisms of angle-closure glaucoma, even in cases of severe corneal opacification. Visualization of the ciliary body is valuable for the diagnosis and management of ocular hypotony, especially in patients with chronic uveitic glaucoma. Anterior segment OCT can provide with substantial imaging information when it comes to the evaluation of the position and length of drainage device tubes^133^ or the filtering bleb in eyes that have undergone relevant operations.^134,135^

## MANAGEMENT OF UVEITIC GLAUCOMA

The therapeutic approach of uveitic glaucoma is individualized and inextricably correlated to the underlying etiology. It comprises the treatment of the ocular inflammation, the underlying systemic disease and of course glaucoma. The inflammation of the eye and glaucoma can be controlled with anti-inflammatory and antiglaucoma agents, respectively. In many cases, management of the inflammatory process contributes to the control of IOP as well. It must be underlined that patients that receive prompt and aggressive anti-inflammatory medication tend to have a significantly better clinical course of glaucoma. In cases that anti-glaucoma medications are inadequate (approximately 25–30%) 1 invasive and surgical options (e.g., laser iridotomy, trabeculectomy, non-penetrating glaucoma surgery, glaucoma drainage device implantation, etc.) are considered.^1,5,6^

### Medical Management

The spectrum of the available medications for IOP lowering has increased dramatically over the last years. The vast majority of these agents can be used to control IOP elevation in uveitic patients, with a few admonitions. This is attributed to the fact that the pressure-lowering effect of most of the topical hypotensive drugs is highly variable in uveitis. It is not uncommon that there might be no response at all or lead to very unstable IOP levels.^74^

Prostaglandin receptor agonists have been thought to have a propensity to increase the inflammatory activity in a small percentage of patients.^136–139^ However, prostaglandin agonists should still be used uveitic eyes cautiously with an ophthalmic history of cystoid macular edema (CMO).^138,140–142^ or herpetic keratouveitis.^143,144^

There is evidence that the efficacy of alpha-adrenergic agonists and prostaglandin agonists may be partially inhibited by the simultaneous administration of non-steroidal anti-inflammatory drugs (NSAIDs). Sponsel et al. reported a partial reduction in the effect of brimonidine in adult patients who were also on oral indomethacin during the same period. The same study showed that the effect of latanoprost remained unchanged.^145^ However, according to a more recent study, the efficiency of latanoprost may be influenced, as it endeavors part of its effect through positive feedback on prostaglandin release.

Consequently, the pressure reduction induced by latanoprost may be partially blocked by the concurrent use of topical Nonsteroidal anti-inflammatory drugs (NSAIDs). It appears that the duration of treatment with latanoprost before starting the use of NSAIDs and the type of NSAIDs administered may play some role.^146^

Notably in eyes with steroid-induced glaucoma and due to the insubordinate nature of the intraocular inflammation, a uveitis specialist can contribute in the reduction of the intensity of steroid treatment by starting the patient on immunosuppressive agents (e.g., methotrexate, azathioprine, cyclosporine, mycophenolate mofetil, and tacrolimus) or even anti-TNF (tumor necrosis factor) agents, such as infliximab or etanercept.^75^ In these cases, it is strongly suggested that the management often optimally includes a Team of physicians including those treating the underlying condition.

Probably, one the most interesting therapeutic prospects of the future is the non-IOP therapy. It has been suggested that TGF-β2 may play a substantial role in the pathophysiology of various types of glaucoma^49,147,148^ TGF-β2 is considered to be involved with the immunosuppressive function of aqueous humor for the maintenance of anterior chamber related immune deviation.^149^ More studies are required to shed more light on the therapeutic indications of TGF-β2 as an anti-inflammatory or immunosuppressant agent in the treatment of glaucoma.

### Filtration Surgery

The non-satisfactory IOP control despite maximum tolerated medical treatment in cases with absence of pupillary block consists an indication for filtration surgery. One of the most critical parameters for a successful filtration operation is to maintain a minimal inflammatory activity before, during and after the time of the surgery.^74^ Although a sufficient control of the uveitic activity for a few months before the surgery is considered to be ideal, in most of the cases filtration surgery is not carried out as an elective procedure. Therefore a preoperative regimen of topical and/or systemic steroids is required to suppress the intraocular inflammatory activity.^150^ Furthermore, an intravitreal 4 mg of triamcinolone or an infraorbital injection of 40 mg of methylprednisolone can be administered at the final stage of surgery.^151^ There are conflicting aspects regarding the use of antiproliferative agents in uveitic glaucoma. There is only a limited number of studies of trabeculectomy with Mitomycin-C (MMC) in uveitic eyes in the current literature, and interestingly, the results indicated no obvious advantage in the control of IOP over 5-fluorouracil.^152–154^ An older retrospective study conducted at the authors’ institution showed a 5-year success rate that did not exceed 30% in uveitic patients that have undergone trabeculectomy without the use of antimetabolites. This rate escalated to 50% by in those who were administered post-operative 5-fluorouracil injections.^155^ Some years later, a prospective study was carried out in the same institution, showing a 5-year success rate of 67% in eyes where a single dose of 5-fluorouracil (25 mg/mL) was given intraoperatively.^156^ Similar findings have been reported in other smaller studies.^152,157,158^ In a more recent study, by Shimizu et al.,^75^ success rates were 82.86% for trabeculectomy with MMC, 62.50% for trabeculectomy, and 75.00% for trabectome. Risk factors that could lead to surgical failure included age less than 45 years, male sex, eyes with non-granulomatous uveitis and post-operative inflammation. About the best-published results of non-antiproliferative trabeculectomy for uveitis, the success rate is 54% after a 5-year interval. It is noteworthy that 78% of these patients needed supplementary antiglaucoma medication.^151^ As it can be inferred the non-satisfying results of unenhanced uveitic trabeculectomy have triggered the investigation of further techniques, such as goniotomy, for the treatment of uveitic glaucoma.

### Glaucoma Drainage Surgery

The complexity of uveitic glaucoma calls for effective surgical alternatives. Generally, it appears that the long-term control of IOP in patients that have undergone a glaucoma drainage device implantation is better than in other categories of glaucoma. This is highly reflected in patients with JIA-related uveitic glaucoma and eyes with other risk factors (e.g., previous filtration surgery failure/s, previous vitreoretinal surgery or aphakia).^74^ Characteristically, Molteno et al. published their results of Molteno tube (Molteno Ophthalmic Limited, Dunedin, New Zealand) implantation in uveitics at 5 and 10 years,^159^ showing very satisfactory long-term results. Other studies investigated the implantation of the Ahmed glaucoma drainage device and the Baerveldt glaucoma drainage device, reporting a 1-year success rate of 94% in 21 uveitic eyes and 2-year success rate of 91.7% in 24 uveitic eyes, respectively.^160,161^

### Cyclodestructive Procedures

It must be underlined that due to the already declined ciliary body, cyclodestruction in uveitic eyes may lead to severe complications, such as a profound IOP drop, phthisis, irreversible anatomical damage and loss of visual function.^74^ A large retrospective study of 263 eyes of several subcategories of insubordinate glaucoma, transscleral diode laser cyclophotocoagulation in uveitics led to higher rates of hypotony in comparison with the other clinical entities.^162^

## CONCLUSION

Uveitic glaucoma comprises a wide spectrum of disorders that very often lead to glaucomatous alterations of the optic nerve and therefore visual field loss. The management of glaucoma in patients with uveitis calls for a thorough diagnostic and therapeutic approach, to achieve adequate control of eye pressure and inflammatory response. In the majority of cases, a multidisciplinary approach is required to ensure a favorable outcome. Prompt and immediate referral to a glaucoma specialist may substantially curtail the risk of blindness in this particular group of patients. Although the treatment modalities for primary open angle glaucoma have significantly improved over the last decades, the management of uveitic glaucoma remains an exceptionally challenging clinical entity. Well-organized randomized control trial studies with a large number of patients are needed to extract significant conclusion regarding the medical and surgical treatment of uveitic glaucoma. These studies could also contribute to defining the optimal primary surgical procedure for this special category of patients. Comprehending the pathogenesis of uveitic glaucoma at a higher level will contribute to the development of more effective therapeutic approaches and strategies to deal with this complicated and potentially blinding disorder.

## LITERATURE SEARCH

This review was based on the literature findings after a MEDLINE/PubMed review (1954 to 2017) using key-words including “uveitic glaucoma,” “ocular hypertension/hypotony in uveitic patients”, “glaucoma and inflammations”, “IOP and uveitis”, “glaucoma and uveitis”. Duplicate articles and foreign language papers were excluded. We also excluded papers that were not related to uveitis or uveitic glaucoma. This procedure led to an overall of 162 original articles that were used to support this study. This study consists of a summary of our current knowledge in the pathogenesis of glaucoma in patients with uveitis.

## ACKNOWLEDGMENTS

Special thanks to Professor Chris D Kalogeropoulos, FEBOphth (Professor of Ophthalmology Head of Ocular Inflammation Service Faculty of Medicine University of Ioannina, GR) for his precious advice and for sharing his experience in the challenging field of uveitic glaucoma.
